# The Role of Natural Products as Inhibitors of JAK/STAT Signaling Pathways in Glioblastoma Treatment

**DOI:** 10.1155/2022/7838583

**Published:** 2022-09-19

**Authors:** Hanieh Fahmideh, Hooriyeh Shapourian, Rasol Moltafeti, Chanour Tavakol, Razieh Forghaniesfidvajani, Hamidreza Zalpoor, Mohsen Nabi-Afjadi

**Affiliations:** ^1^Department of Microbiology, School of Medicine, Shahid Beheshti University of Medical Sciences, Tehran, Iran; ^2^Department of Immunology, Faculty of Medicine, Isfahan University of Medical Sciences, Isfahan, Iran; ^3^Pediatric Department of Bo-Ali Hospital, Ardabil University of Medical Sciences, Ardabil, Iran; ^4^School of Medicine, Tehran University of Medical Sciences, Tehran., Iran; ^5^Network of Immunity in Infection, Malignancy & Autoimmunity (NIIMA), Universal Scientific Education & Research Network (USERN), Tehran, Iran; ^6^Shiraz Neuroscience Research Center, Shiraz University of Medical Sciences, Shiraz, Iran; ^7^Department of Biochemistry, Faculty of Biological Science, Tarbiat Modares University, Tehran, Iran

## Abstract

The permeability of glioblastoma, as well as its escaping the immune system, makes them one of the most deadly human malignancies. By avoiding programmed cell death (apoptosis), unlimited cell growth and metastatic ability could dramatically affect the immune system. Genetic mutations, epigenetic changes, and overexpression of oncogenes can cause this process. On the other hand, the blood-brain barrier (BBB) and intratumor heterogeneity are important factors causing resistance to therapy. Several signaling pathways have been identified in this field, including the Janus tyrosine kinase (JAK) converter and signal transducer and activator of transcription (STAT) activator pathways, which are closely related. In addition, the JAK/STAT signaling pathway contributes to a wide array of tumorigenesis functions, including replication, anti-apoptosis, angiogenesis, and immune suppression. Introducing this pathway as the main tumorigenesis and treatment resistance center can give a better understanding of how it operates. In light of this, it is an important goal in treating many disorders, particularly cancer. The inhibition of this signaling pathway is being considered an approach to the treatment of glioblastoma. The use of natural products alternatively to conventional therapies is another area of research interest among researchers. Some natural products that originate from plants or natural sources can interfere with JAK/STAT signaling in human malignant cells, also by stopping the progression and phosphorylation of JAK/STAT, inducing apoptosis, and stopping the cell cycle. Natural products are a viable alternative to conventional chemotherapy because of their cost-effectiveness, wide availability, and almost no side effects.

## 1. Introduction

Compared to other types of brain tumors, glioblastomas are very aggressive. Glioblastoma is a primary brain tumor that arises from glial tissue and is among the most common and deadly of all brain tumors due to its high permeability, immune system evasion ability, and molecular heterogeneity [1]. Despite many attempts, it has proven to be highly resistant to conventional treatment, and its recurrence has made many treatments seem ineffective. Among the reasons for resistance to treatment are the blood-brain barrier (BBB) as well as intratumor heterogeneity [2, 3]. Abnormal tumor cells have some features that complicate tumors and make them ineffective to treat. Among these are high proliferation rates, escape mechanisms from the immune system, resistance to programmed cell death (apoptosis), angiogenesis, and maintenance of cell signaling to induce repetitive immortality, invasive activity, and metastasis [4]. If any of the above mechanisms is stopped, there will be advancements in cancer treatment. The biological cell survival abilities of tumors can be caused by genetic mutations, epigenetic changes, and overexpression of oncogenes [5]. Tumor development is caused by many factors, one of the most important factors is the oncogenic signaling pathway. In this field, many signaling pathways have been identified. One of the most important of them is the Janus tyrosine kinase (JAK) converter and transcription signaling (STAT) activator pathway, which is closely related and manages the replication, antiapoptotic, angiogenic, and immunosuppressive functions in tumor microenvironment [6–8]. It is believed that this route is responsible for the main source of tumor formation and resistance to treatments [2]. Therefore, blocking the abilities of this signaling pathway is seen as a potential approach to treat glioblastoma. In addition to conventional treatments, a new approach called “natural products” is being considered by researchers [5, 9]. The therapeutic effects of these bioactive compounds, which are mainly found in plants, have yet to be independently verified in the case of cancer [5, 10–15]. Nevertheless, studies and preclinical data have shown that natural compounds in plants can affect the signaling pathways and proliferation of malignancies in humans [11]. They can also strengthen and sensitize the immune system against cytotoxic agents. Furthermore, studies have found that this method has fewer side effects than conventional chemotherapy methods, and it is less toxic to organs such as the liver, heart, and kidneys [16]. The current research on this topic has been reviewed, and some of these bioactive elements have been excerpted in relation to their ability for affecting JAK/STAT signaling pathways. The purpose of this study is to collect and suggest the preventive factors and possible natural treatments for glioblastoma.

## 2. JAK/STAT Signaling Pathways and Glioblastoma

JAK/STAT are closely related to each other and together can promote growth, proliferation, survival, inflammation, invasion, blood vessel formation, and progression of multiple tumors [17]. The four main members of JAK are JAK1, JAK2, JAK3, and TYK2. STATs fall into seven major groups: STAT1, STAT2, STAT3, STAT4, STAT5a, STAT5b, and STAT6 [18–20]. The STAT protein, which is dormant in the cytoplasm, is the initiator of this pathway [21]. Following receptor dimerization, JAK proteins are activated. It is important to note that cytoplasmic domains of receptors contain tyrosine residues. After phosphorylation of these residues, JAK is activated to transphosphorylate the remaining tyrosine residues to form a binding site for the SH-2 domain of STAT proteins. As STAT molecules are transferred from the cytoplasm to the nucleus, they phosphorylate target genes by binding to DNA regulatory elements (DREs) [22, 23]. A change in this signaling pathway can influence pathological processes due to its important role in the regulation of biological processes because a malfunctioning of Janus kinase, JAK signal converter, and STAT signaling can lead to tumor formation [17]. Glioblastoma is a prevalent primary brain tumor in humans, and a highly infiltrative and extremely aggressive astrocytoma, which is characterized by resistance to apoptosis by radiation and chemotherapeutic treatments [7, 24]. Several studies indicate that JAK/STAT signaling pathways are involved in the progression, migration, and invasion of glioblastoma. For instance, a study found that glioma cells and other immune cells secrete IL-8, which promotes tumor migration, invasion, and mesenchymal transition by activating STAT1/hypoxia-inducible factor-1*α* (HIF-1*α*)/Snail pathway [25]. In glioblastoma, STAT5 signaling plays a role in proliferation and invasion, which are linked with tumorigenesis [26, 27]. Additionally, patients with glioblastoma have constitutively activated STAT3 and secreted IL-6 levels that are correlated with tumor grade [24]. The glioma cell-secreted granulocyte macrophage colony-stimulating factor (GM-CSF) activates STAT5 signaling in myeloid-derived suppressor cells (MDSCs), so Bcl2a1 is expressed and IRF8 is downregulated and they inhibit apoptosis and stimulate proliferation, respectively. Among all STAT family members, STAT3 has the most comprehensive oncogenic activity and immune suppressive role in glioblastoma. In malignant gliomas, aberrant STAT3 signaling is primarily the result of dysregulated upstream pathways, which drive proliferation, neovascularization, apoptosis resistance, and immune escape [2]. The interactions of tumor cells, reactive astrocytes, and microglia in glioblastoma lead to high-level expression of TGF-*β* and IL-10, which promote a positive feedback loop for STAT3 signaling and generate an immunosuppressive cytokine milieu [28]. A number of cellular populations, including reactive astrocytes, express IL-6 within the TME, which increases STAT3 signaling via JAK activation [29]. Upregulation of JAK/STAT gene targets, such as cytokines, cytokine receptors, and JAKs, is associated with a poor prognosis in classical glioblastoma [30]. The signaling pathway with phosphorylation of JAK2/STAT3 is associated with the management and enhancement of cancer cell proliferation, as well as resistance to radiation therapy that is one of the key roles of STAT signaling [31]. To inhibit STAT signaling, proteins like protein tyrosine phosphatases (PTP), cytokine signaling suppressors (SOCS), and active STAT protein inhibitors (PIAS) act as endogenous inhibitors [32]. So, targeting this pathway can suppress the expression of target genes that control essential cell functions and help to treat cancer by causing cell death (apoptosis) [33]. Modulating and controlling STAT can be an effective strategy for protecting cells and tissues from malignant tumors [34].

Research on natural products has been boomed over the past few years, and the evidence that improper activation of STATs may cause malignancies in humans has prompted researchers to respond positively to this signaling pathway's link to natural products [35]. Especially, since natural agents have demonstrated strong anticancer activity across a wide range of mechanisms and recent studies suggest phytochemicals can inhibit the JAK/STAT pathway and growth of cancer cells [5].

## 3. Natural Products with Inhibitory Effects on JAK/STAT Signaling Pathways

**Figure 1:**
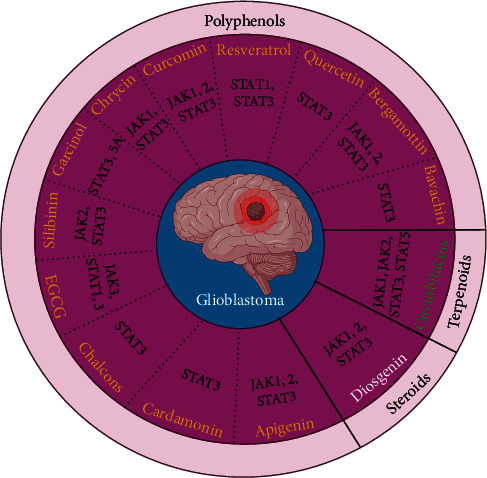
Polyphenols, steroids, and terpenoids that can lead to glioblastoma treatment by inhibiting JAK-STAT signaling pathways.

Since phytochemicals have been studied in a variety of cancers, some of these natural compounds have inhibited countless inflammatory pathways. By reducing the production of certain cytokines, some of these compounds are capable of inhibiting the STAT3 phosphorylation pathway and others act directly as inhibitors of JAK [36, 37]. In other research, the factors that control the SH2 domain and limit STAT dimerization have been investigated [38]. Several plant groups have been studied, including phenols (including resveratrol, curcumin, bergamottin, capillarisin, emodin, garcinol, cardamonin, casticin, and apigenin that inhibit JAK1,2 as well as STAT3), steroids (including diosgenin, ergosterol peroxide, and guggulsterone that inhibit JAK1/JAK2 and DNA binding activity as well as STAT3), and terpenoids (including cucurbitacin, andrographolide, betulinic acid, cryptotanshinone, celastrol, oridonin, and alantolactone that inhibit JAK1 and JAK2 as well as STAT3 and STAT5) and these studies have yielded promising results for cancer prevention and therapy [5]. In the next section, we described the mechanisms and anticancer effects of some phytochemicals that act as inhibitors of the JAK/STAT signaling pathways by focusing on their therapeutic roles in glioblastoma (Figure 1).

### 3.1. Phenolics and Polyphenols

There are over 8,000 components of these plant compounds in nature, all of them contain hydroxyl groups and aromatic rings. These metabolites are water-soluble and exist in various vegetables and fruits. They can also control various biochemical and pharmacological effects, such as anti-inflammatory mechanisms, immune-modulating, and antioxidant effects, as well as regulation of some signaling pathways [5, 39].

#### 3.1.1. Resveratrol

Resveratrol is an anti-inflammatory phytoalexin with antioxidant properties [40]. Its chemical and anticancer effects have been shown in multiple studies. Resveratrol can be found in peanuts, grapes, and berries, also known as *trans*-3,5,40-trihydroxyacetylbene. In a number of studies, it has been demonstrated that resveratrol administration is effective in glioblastoma treatment through inhibition of cancer cell proliferation, migration, and viability via inhibition of various molecular pathways [41]. The apoptotic and amplifying activities of this compound are related to the inhibition of JAK/STAT signaling, as it can prevent STAT1 phosphorylation by inhibiting JAK and STAT3, which reduces the activity of antiapoptotic genes and induces tumor cell death. Src/STAT and JAK/STAT pathways can both be blocked by resveratrol, that is, demonstrating its antitumor activity [5, 42, 43]. Recent research has shown that resveratrol can increase ROS generation and induce oxidation-related cellular lesions in U251 cells (malignant glioblastoma cell lines) by activating a ROS-related mitochondrial signaling pathway [44]. On glioblastoma cell lines LN18 and U87, Song et al. indicated that resveratrol reduced epithelial to mesenchymal transition (EMT), expression of *β*-catenin, and decreased the expression of stem cell markers (Twist, Snail, MMP-2, MMP-9, Slug, and Smad) [45] (Table 1).

#### 3.1.2. Curcumin

Curcumin, a member of the ginger family (*Zingiberaceae*) is a diarylheptanoid compound, which is derived from turmeric's rhizome. For many years, it has been used to treat chronic illnesses such as neoplastic diseases. There has been extensive evidence that curcumin has anti-inflammatory, antitumor, and antioxidant properties. Furthermore, it improves drug resistance in cancer therapy, so its consumption may overcome the resistance to gefitinib or erlotinib in humans with non-small-cell lung cancer (NSCLC) as a potential epidermal growth factor receptor tyrosine kinase inhibitor (EGFR-TKI) [46, 47].

By inhibiting tumor microenvironment mechanisms such as inflammation, angiogenesis, and metastasis, it can prevent the progression of malignant tumors. Additionally, it is capable of influencing and inhibiting molecular signaling mechanisms [48]. The anticancer activity of curcumin has been linked to its ability to modulate oncogenes (egr-1, c-Myc, Bcl-XL, NF-*κ*B, and p53), transcription factors (NF-*κ*B, STAT3, and AP-1), and protein enzymes (COX and LOX) [49–51].

There are numerous studies in which it has been shown that curcumin inhibits STAT3 through induction of ROS or RANK gene activity in glioblastoma cells, or by suppressing JAK1, 2/STAT3 phosphorylation through downregulation of MMP-9, c-Myc, Ki-67, and Snail, suppressing cell proliferation, migration, and invasion by inducing G2/M cell cycle arrest [52–56] (Table 1).

**Figure 2:**
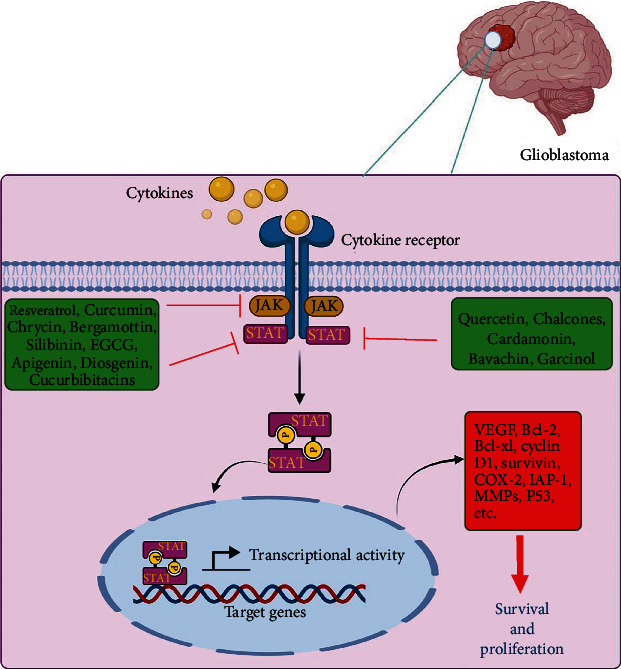
Inhibitory effects of natural products on the JAK/STAT signaling pathway in glioblastoma.

So, by suppressing STAT3, curcumin disrupts the JAK/STAT signaling pathway (Figure 2). Various studies have shown that this plant compound can suppress proliferative and invastive proteins as well [57]. Some preclinical trials have shown that curcumin increases the effectiveness of existing chemotherapeutic agents, which is a great benefit [58].

#### 3.1.3. Bergamottin

Bergamottin is a furanocoumarin with anti-inflammatory and antioxidant properties isolated from grapes. This compound is found in bergamot, grapefruit, and lemon. Furanocoumarin is primarily used by plants to defend themselves against predators and is considered a natural pesticide. Additionally, it acts as an inhibitor of some cytochrome enzymes, such as P450 [59, 60]. By regulating the signal transducer and activating the STAT3 transcription signaling pathway, which is linked to tumor progression, this combination can negatively regulate the cell cycle and activate apoptosis. This suppression is achieved by inhibiting phosphorylation of activated kinases of JAK1, JAK2, C-Src, and SHP-1 and suppressing STAT3 and its downstream products including Bcl-2, Bcl-XL, cyclin D1, COX-2, IAP-1, survivin, cyclin D1, and VEGF [5, 56, 61, 62].

Also, it was revealed that treatment of human glioma cells with bergamottin significantly inhibited wound-healing migration and Matrigel invasion of human glioma cells, relative to untreated cells by inhibition of EMT, JNK, PI3K, Akt, NF-*κ*B,, STAT3, Rac1, and mTOR kinases, and MMP-9 production in A549, U87, and U251 cell lines [63, 64] (Table 1).

#### 3.1.4. Bavachin

Bavachin inhibits STAT3 transcription by acting as a phytoestrogen. This compound is obtained from plants such as *Psoralea corylifolia*. By inhibiting NF-*κ*B and IL-6-induced STAT3 activity and activating caspases 3 and 9, it can stimulate apoptosis [65]. Bavachin also modulates the expression of phorbol-12-myristate-13-acetate-induced protein 1 (PMAIP1, alternative known as Noxa) and p53 which play a role in tumor cells' survival [5, 66].

Bavachin also suppresses the inflammation caused by LPS, which causes the release of (nitric oxide) NO, (prostaglandin E2) PGE 2, as well as IL-6 (produced by macrophage M1) and (NOD-, LRR-, and pyrin domain-containing protein 3) NLRP3 (in macrophages). In response to the release of NLRP3, the proinflammatory cytokine IL-1*β* is activated, which contributes to pyroptosis [67–69]. Bavachin also decreases HIF-1*α* activity, the main oxygen sensor within cells, under hypoxia in a concentration-dependent manner and reduces HIF-1-regulated transcription of genes related to energy metabolisms, such as glucose transporter type 1 (GLUT1) and hexokinase 2 [70] (Table 1).

#### 3.1.5. Epigallocatechin Gallate (EGCG)

Catechins in green tea act as potent antioxidants and prevent the progression of tumors by acting as antiangiogenic agents. By affecting the expression of cell cycle regulatory proteins, inhibiting JAK3/STAT3 signaling, and activating lethal caspases, this compound induces apoptosis and cell proliferation as an EGFR-TKIs. In this line, EGCG, also known as epigallocatechin-3-gallate, inhibits the EGFR dimerization and its activation or even binds to EGF to inhibit the EGFR phosphorylation. Moreover, EGCG suppresses glioblastoma development by telomere shortening, elevating DNA damage through phosphorylation of *γ*-H2AX histone, micronuclei, and telomere dysfunction [51, 71].

In addition, it inhibits carcinogenesis by affecting a wide range of signaling pathways, including Wnt, MAPK, Notch, and PI3K/Akt. The low cost and high immunity of this compound make it a great candidate for preventing cancer. This nontoxic natural agent can be used to treat malignancies in humans, either alone or in combination with other treatments [72, 73].

Furthermore, EGCG with quercetin can block the JAK/STAT pathway, ultimately making STAT1 and STAT3 inactive in cholangiocarcinoma (CCA) cells [71]. A dose-dependent reduction of phosphorylated STAT1/3 was observed when this combination was administered. In addition, this study reaveled that CCA cells' proliferation and migration were also impaired [74]. In a colorectal carcinoma microenvironment and in a PDX mouse model, administration of curcumin and EGCG together inhibited angiogenesis via the Janus kinase/STAT3/IL-8 pathway [75]. In a study, Grube et al. indicated that after 500 *μ*M concentration of EGCG, strong induction of autophagy and apoptosis was observed in glioblastoma cultures, whereas its 100 nM concentration leads to elevate accumulation of autophagic vacuoles and reactive oxygen species production as a stress response in the first 12 h of treatment (Table 1). This data indicates that although green tea may have chemopreventive properties, it is not directly cytotoxic [76].

#### 3.1.6. Chalcones

Chalcones are phenolic compounds in the flavonoid family. Among the fruits and vegetables that contain chalcones are oranges, strawberries, potatoes, and bean sprouts, as well as some spices, like licorice. Through their antioxidant, cytotoxic, and cell signaling modulatory properties, these phytochemicals are involved in the reduction of malignant tumors progression. In addition, this phenolic compound induces apoptosis [5]. Unsaturated flavonoids induce apoptosis by inhibiting STAT3 phosphorylation and activating caspases 8 and 9. As a result of releasing ROS and changing the mitochondrial membrane potential, cytochrome C is released and the death process is initiated [77]. Researchers have studied cardamonin (CAR), a chalcone isolated from plants of the Zingiberaceae, Asteraceae, Piperaceae, Polygonaceae, and many other families for its health benefits, including anti-inflammatory, antioxidant, and antineoplastic properties [78]. According to studies, the inhibition of STAT3 activation may also be involved in cardamonin-induced apoptosis. In glioblastoma stem cells, Wu et al. found that CAR inhibited STAT3 phosphorylation, blocked STAT3 nuclear transport, and attenuated the expression of downstream genes including VEGF, survivin, Bcl-XL, and Bcl-2 [79]. A similar effect of CAR on STAT3 signaling has also been reported in prostate cancer cells [80] (Table 1). Hence, chalcones could be considered potential anticancer drug compounds due to their multitarget action.

#### 3.1.7. Garcinol

Garcinol has been noted for its antioxidant and cancer-fighting properties, like other phytochemicals. The leaves and fruits of *Garcinia indica* contain biologically active compounds, and several signal pathways are effectively inhibited by garcinol [81]. Furthermore, it inhibits STAT3 acetylation and dimerization and negatively affects the protein's ability to bind to DNA. Studies have shown that the basic mechanism of garcinol is the induction of apoptosis, which is facilitated by reducing the NF-*κ*B pathway [82]. In order to investigate the molecular mechanism of garcinol's action on pancreatic cancer cells, signaling molecules involved in apoptosis (X-IAP, cIAP, caspase 3/9, and PARP cleavage) were targeted, as well as NF-*κ*B, VEGF, IL-8, and PGE2, which involve in chemoresistance in pancreatic tumors. The antiproliferative, proapoptotic, antimetastatic, and antiangiogenic effects of garcinol in pancreatic cancer cells were significantly enhanced relative to those in untreated cells [83]. Study results showed that garcinol inhibited both total and phosphorylated STAT3 in breast, pancreatic, and prostate cancer cell lines and reduced cell invasion in these cancer cell lines in a dose-dependent manner [84]. A study by Liu et al. showed that garcinol inhibited proliferation, invasion, and migration in glioblastoma U-87 MG and GBM8401 cells dose dependently, which was mediated by increasing the hsa-miR-181d/STAT3 and hsa-miR-181d/STAT5A ratios in glioblastoma cells [85]. Garcinol reduced glioblastoma tumor growth in an immunocompromised mouse model by reducing STAT3/STAT5A expression, enhancing the Bax/Bcl-XL apoptotic ratio, and downregulating the Ki-67 proliferation index, in vivo [86] (Table 1).

#### 3.1.8. Silibinin

Silibinin is a nonsteroidal anti-inflammatory drug that targets inflammation and positively affects cellular and noncellular components [87]. It is a plant compound derived from milk thistle that was previously used for the treatment of chronic liver disease. Moreover, it is an antioxidant/anti-inflammatory flavonoid that offers an appealing strategy for the treatment of cancer [88]. Several studies have demonstrated the benefits of this phytochemical in combination with conventional chemotherapy treatments, including a reduction in adverse neurological, cardiac, and renal effects [5]. In the presence of silibinin activity, JAK2 inhibitors reduced STAT3 phosphorylation but did not inhibit it. On the other hand, inhibition of JAK1 completely inhibits STAT3 phosphorylation and activates caspase 9 and 3, which cause apoptosis [89]. The downregulation of miR-21 by silibinin appears to activate the p53 pathway, as well as several genes related to intrinsic and extrinsic apoptosis. In glioblastoma cells, it has been reported that miR-21 inhibition by antisense oligonucleotides increased caspase activation and cell death (Table 1). Together, these results suggest that silibinin might induce apoptosis and cell cycle arrest in part by inhibiting miR-21 and miR-155 as oncomiRs and may be able to have a positive impact on eliminating human malignancies [89, 90].

#### 3.1.9. Chrysin

It has been demonstrated that chrysin is a naturally occurring flavonoid found primarily in propolis and honey. As a result of its beneficial biological properties (anti-inflammatory, antitumor, antioxidant, and antiestrogen), this compound, known as 5,7-dihydroxyflavone, is included in the list of natural products for anticancer. The compound has drawn the most attention due to its low toxicity, which inhibits angiogenesis, metastasis, and tumor growth [91, 92]. This combination has inhibited the signaling pathway of Akt and STAT in preclinical trials [5]. Based on the data, chrysin appears to be able to downregulate soluble IL-6 receptor (IL-6R), glycoprotein 130 (gp130), phosphorylated JAK1, and STAT3 levels and VEGF in human umbilical vein endothelial cells (HUVECs) [93]. Chrysin exerts antitumor activity via direct interaction with multiple molecular targets and modulation of signal transduction pathways involved in cellular metabolism (AMPK/Akt/ERK/PPAR) and inflammation (NF-*κ*B, P38/MAPK, TBK1, and Wnt/*β*-catenin) [94] (Table 1). During chyrsin treatment, glioma cells were arrested in the G1 phase due to increased P21 (waf1/cip1) and activation of P38-MAPK [95]. Chrysin displayed greater antiglioblastoma activity in GBM8901 cells compared to other compounds (PWE, pinocembrin, and tiliroside), and it reduced growth in a time-dependent manner from 25 to 100 *μ*M in GBM8901 cells. However, the chrysin compound did not cause damage to other glial cell lines; this is suggesting that it might be able to display specific antiglioblastoma properties without damaging normal cells [96].

#### 3.1.10. Apigenin

This flavonoid can be found in fruits (e.g., citrus and apple), vegetables (e.g., parsley and celery leaves), and some medicinal plants (chamomile, thyme, oregano, lemon balm, and yarrow). It has been proven to have antitumor properties and is considered by researchers. Apigenin inhibits angiogenesis by reducing the glucose uptake of cancer cells and inhibiting adhesion molecules [97]. The anticancer activity of this compound is through inhibition of JAK1/2 and STAT3 (Figure 2, Table 1). This compound induces apoptosis, reduces Mcl-1 and Bcl-XL, and thus inhibits STAT3 phosphorylation [98]. Treatment with apigenin caused G2/M arrest in glioblastoma cells and decreased levels of Akt, mTOR, ERK, STAT3, and S6K proteins [99, 100]. Researchers found that apigenin reduced survival, growth, proliferation, and migration of rat C6 glioma cells by altering their cytokine profiles, which are important for regulating the immune response [101] (Table 1). Taking the drug in combination with other flavonoids also led to synergistic activity by decreasing MMP-2 expression and increasing fibronectin, laminin, and glial fibrillary acidic protein (GFAP) expressions [102]. By regulating the levels of TNF-*α*, caspases, and apoptotic proteins, apigenin with hydroxygenkwanin provided enhanced antiglioma activity [103].

#### 3.1.11. Quercetin

Quercetin (chemically known as 3,3′,4′,5,7-pentahydroxy flavone) is a polyphenolic flavonoid found in a variety of fruits and vegetables [104]. Because of its anti-inflammatory, antioxidant, and antitumor effects, quercetin has been extensively studied in vitro and in vivo in multiple cancer models including lung, gastric, cervical, breast, prostate, and colon [105–108]. Quercetin has the ability to impact apoptosis and arrest phase G1 cell cycle in tumor cells, through its interaction with cell cycle regulators, including cyclin-dependent kinase- (CDK-) 4 and cyclin D1, activating p53, cytochrome c release, and also inducing caspase 9 and caspase 3 release [109]. Furthermore, quercetin can be viewed as a potential multitarget agent and a potential PI3K inhibitor [110] (Table 1). By chemoprotecting normal cells from chemotherapy and radiotherapy, this phytochemical could provide a significant advantage for anticancer treatment [106]. In a variety of cellular and animal models as well as in humans, quercetin modulates signaling pathways and gene expression to exert antioxidant, anti-inflammatory, and antitumor properties [111]. The anticancer effects of this natrual drug for the treatment of glioblastoma may occur mainly through the regulation of PI3K/Akt/mTOR signaling pathways, IL-6/STAT3 signaling pathways, modulation of apoptosis-related proteins, alteration of intracellular pH (pHi), and MMP-2/9 and fibronectin expressions [106] (Figure 2).

### 3.2. Terpenoids

Compounds called isoprenoids are secondary plant metabolites that are abundant in nature. Several terpenoids have been used to treat cancer, including taxol [112]. Phytochemicals such as this combination have antifungal, antimicrobial, antiviral, antispasmodic, and immunomodulating properties. Research has shown that terpenoids inhibit the growth of cancer cells without causing any toxicity to normal human cells. It has been identified that various types of these compounds (cucurbitacins, andrographolide, ryptotanshinone, nimbolide, etc.) can inhibit JAK/STAT signaling in human malignancies [5]. Here, we described cucurbitacin's effects on glioblastoma treatment by targeting JAK/STAT signaling pathways, while it seems that future investigations are required to determine the therapeutic effects of other terpenoids (e.g., andrographolide, ryptotanshinone, and nimbolide) on glioblastoma by inhibiting JAK/STAT signaling pathways.

#### 3.2.1. Cucurbitacin

It is a four-ring triterpene compound that is highly oxidized. It can inhibit some signaling pathways (JAK/STAT3, Wnt, PI3K/Akt, and MAPK) and has strong anticancer activity. The mechanism of its action is antiproliferation, inhibition of growth and mutation, and increase of cell cycle arrest [113]. Analogues of cucurbitacins range from cucurbitacin A to cocorbitacin T. The cucurbitacin E compound inhibits the cell cycle and induces apoptosis in T24 cells (human bladder carcinoma cell line). It is believed that this cucurbitacin plays a role in halting the G2/M phase of the cell cycle by activating caspases 8, 9, 3 and Fas/CD95 and inhibiting STAT3/P53/P21 signaling [114]. Cucurbitacin B inhibits STAT3 activation and Raf/MEK/ERK signaling pathways in K562 cells (leukemia cell line) [115]. According to a study, cucurbitacin I markedly decreased p-JAK1, p-JAK2, p-STAT3, and p-STAT5 levels and inhibited proliferation of glioblastoma cells [116] (Figures 1 and 2). Curcurbitacin I (JSI-124) potently inhibits VEGF-induced JAK2 and STAT3 activation. Coculturing HUVECs with glioblastoma cells increased their migration rate significantly. However, coculturing JSI-124-treated glioblastoma cells and HUVECs led to a marked decrease in HUVEC migration. This compound can inhibit HUVEC tubular formation induced by U87MG cells [117, 118] (Table 1). It seems that cucurbitacin will be increasingly used in the near future to target oncogenic pathways therapeutically.

### 3.3. Steroids

Steroids come from phytosterols, a class of compounds similar to cholesterol but differing in the number of hydrocarbon chains [119]. This phytochemical has been shown to have beneficial effects on diabetes and heart disease. According to new research, phytosterol consumption is directly connected to cancer prevention. The human body can be protected by them, and they can prevent cell proliferation and metastasis [119]. The compositions in these compounds affect hormone-dependent endocrine glands to exert their antitumor effects [120]. The next paragraph is about one drug from this phytochemical family.

#### 3.3.1. Diosgenin

The compound in subject is a steroid saponin found in legumes, potatoes, and certain vegetables [121]. Also, fenugreek contains large amounts of diosgenin. In both preclinical and clinical studies, the therapeutic effects of fenugreek (antidiabetic, antihyperlipidemic, antiobesity, anticancer, anti-inflammatory, antioxidant, antifungal, and antibacterial) have been demonstrated [122]. Both in vitro and in vivo studies have shown that dioscin (the structural analog of diosgenin) is effective against glioblastoma. Dioscin treatment increased apoptosis, ROS generation, DNA damage, and arrest of the S phase cell cycle in C6 glioma cells [123]. STAT3 is affected by JAK1, c-Src, and JAK2 activation; these pathways are also suppressed by diosgenin [124] (Figure 2, Table 1). A decrease in cyclin D protein is caused by diosgenin, which inhibits cell proliferation and stops G0/G1 phase [125]. A study found that diosgenin decreased STAT3-regulated gene expression and inhibited hepatocellular carcinoma (HCC) proliferation. Diosgenin appears to be a novel STAT3 activation pathway inhibitor with potential application to treatment of HCC and other cancers [124].

## 4. Some Other Kinds of Natural Products or Diets Which Might Be Useful to Reduce Glioblastoma

### 4.1. Extra Virgin Olive Oil and Other Oils

Nowadays, more attention is paid to the use of natural products for the treatment of glioblastoma, but there are still many natural compounds with promising antioxidant/anti-inflammatory properties through the JAK-STAT pathway that have been overlooked.

There are several studies that examined the effects of some kinds of natural products as useful diets to reduce glioblastoma complications. A study by Lamy et al. on olive oil compounds and their effect on glioblastoma cells has revealed that four compounds contained in extra virgin olive oil including hydroxytyrosol (HT), oleuropein (OL), oleic acid (OA), and tyrosol (Tyr) can inhibit tumor necrosis factor-*α-* (TNF-*α-*) induced expression of cyclooxygenase-2 (COX-2) in a U-87 MG cell line model as a human glioblastoma cell. Furthermore, among these combinations, Tyr and OA could significantly inhibit TNF-*α*-induced COX-2 gene and protein expression, as well as prostaglandin E2 (PGE2) secretion, prevent TNF-*α*-induced JNK and ERK phosphorylation and also reduce human brain microvascular endothelial cells (HBMECs) migration [127]. Another examination of the systemic lupus erythematosus (SLE) model in mice demonstrated that dietary extra virgin olive oil ameliorates kidney injury through MAPK activation and suppression of JAK/STAT and NF-*κ*B pathways [128]. Based on traditional Persian medicine, citral, a major component of lemon balm essential oil, induces apoptosis in GBM cells that express active multidrug resistance-associated protein 1 (MRP1) [129].

Currently, a natural combination of the wild olive tree which is called acebuche (ACE) in Spain is being suggested for attenuating ocular oxidative stress induced by arterial hypertension. This study has revealed that ACE oil-enriched diet reduced nicotinamide adenine dinucleotide phosphate (NADPH) oxidase activity in gene and protein levels and improved alterations in bioavailability of nitric oxide and antioxidant enzyme profile in the retinas of hypertensive mice [130]. The use of a diet with extra virgin olive oil containing Hydroxytyrosol-acetate (HTy-Ac) has been shown to reduce serum synovial and cartilage biomarkers such as cartilage oligomeric matrix protein (COMP) and MMP-3 through the activation of the nuclear factor- (erythroid-derived 2-) like 2/heme oxygenase (Nrf2/HO-1) pathway and the inhibition of relevant signaling pathways like JAK-STAT, MAPKs, and NF-*κ*B in a model of collagen-induced arthritis (CIA) in mice [131]. However, approximately there is no information about testing the ACE potential therapeutic properties for glioblastoma and it remains elusive. Therefore, it is necessary to conduct preclinical and clinical studies in this field.

### 4.2. *Withania somnifera* and Withaferin-A

In addition, there are other interesting plants with properties in regulation of the JAK/STAT pathway that could be used to treat glioblastoma. In diverse preclinical cancer models, *Withania somnifera* as a magic plant and its extracts like withaferin-A (WA), indicated anti-inflammatory activities and regulated several signaling pathways such as JAK/STAT, NF-*κ*B, and AP-1. The compound is suggested for treating cancer patients with drug resistance. However, clinical trials are testing this natural product for cancer therapy [132]. The development of steroidal lactone, WA as an anticancer agent which is obtained from the roots and leaves of withania somnifera, may provide a novel approach for treating glioblastoma since it modulates several oncogenic pathways simultaneously. Some WA properties which are collected from studies on glioblastoma are antiproliferative activities and modulating MAPK, JAK/STAT, and Akt/mTOR signaling, induction of apoptosis and cell cycle arrest, modulation of HSP60/HSP70 chaperones, and effect on DNA damage and repair mechanisms [133–136].

### 4.3. Blueberries and Malvidin

The benefits of blueberries, rich in anthocyanins, have gained considerable attention as functional foods. The major constituent of blueberry, malvidin, has been shown to abolish the JAK/STAT3 pathway, downregulate cell proliferation, and induce mitochondrial-mediated apoptosis in a hamster model of oral oncogenesis. In addition, the combination of S3I-201, a STAT3 inhibitor, with blueberry and malvidin was more effective in STAT3 inhibition respective to the single agent [137]. The compound resveratrol, found in blueberries, inhibits the growth of cells, induces apoptosis, stops G0/G1-phase cell cycle arrest, and blocks STAT3/JAK2 signaling [138]. Malvidin could inhibit EMT in glioblastoma cells (U-87MG) by signaling through TGF-*β*/SMAD2 [139]. It sounds that berries and their combinations are precious sources for glioblastoma treatment. But, despite the above studies, it is still essential to conduct more precise clinical studies on the effect of blueberries compounds and malvidin on glioblastoma cells through the JAK/STAT pathway.

### 4.4. Vitamin C and E

Other natural products with antioxidant/anti-inflammatory properties that can be used for the treatment of glioblastoma are vitamins which have received more attention today [140]. The results of experiments performed on glioblastoma cell lines and tumor xenografts suggest that high doses of ascorbate (vitamin C) induce cytotoxicity and oxidative stress in malignant cells selectively in comparison to normal cells. At present, only a few numbers of studies are available that test ascorbate as a potential therapeutic agent for glioblastoma patients. Nevertheless, the combination of pharmaceutical ascorbate with radiation and temozolomide has the potential to improve the patient's treatment performance and quality of life [141]. It has been shown by Yiang et al. that methotrexate (MTX), a clinical drug for cancer treatment, alone or in combination with vitamins C and E inhibits the growth of GBM cells [142]. Vitamin C appears to regulate JAK/STAT, TRAIL, TGF/SMAD, and microRNAs in different cancers according to scattered scientific evidence [143]. It seems that antioxidant vitamins such as vitamin C and E could be used as early, unconventional, and inexpensive therapy for the treatment of glioblastoma. In order to confirm these findings, larger cohorts and placebo-controlled trials should be administered.

## 5. Future Directions and Conclusions

The median survival rate remains bleak for glioblastoma, as the most common malignant brain tumor in adults. Even though numerous attempts have been made, very few FDA-approved drugs are available for its treatment, and they do not work for everyone [144]. Chemotherapy, which significantly destabilizes the cell metabolism and signaling network, can also lead to drug resistance in glioblastoma treatment [8, 145]. The majority of drugs used in cancer therapy are natural products or their structural relatives. Combinatorial chemistry and high-throughput screening technologies are used by the pharmaceutical industry to obtain these drugs [146, 147]. Thus, more subtle use of nature-derived templates combined with organic chemistry will be much more likely to produce selective analogs [148]. Glioblastoma patients have failed most chemotherapy trials partly due to poor drug penetration through the BBB. Consequently, natural products may alter the function of BBB components and therefore alter their permeability [149]. An understanding of glioblastoma biology and the microenvironment may allow researchers to develop more precise target-specific approaches using natural products (raw or modified formulations) to prevent and treat glioblastoma while minimizing severe side effects and off-target risks associated with conventional chemotherapies as well as improving treatment efficacy. Nevertheless, the difficulties associated with the development of these anticancer agents include poor solubility, resistance to development, and damaging side effects. To overcome these difficulties, a nanotechnology platform has become successful in recent times in providing “nano or other modified natural products” and providing a new dimension and face to natural products for the treatment of cancer [150, 151].

Targeting JAK/STAT signaling pathways has been established to be a practical therapeutic approach for a variety of cancers, including glioblastoma. This review discussed multiple natural products with therapeutic effects on glioblastoma by targeting JAK/STAT signaling pathways. These natural products can be combined with other common JAK/STAT inhibitors to reduce their required dosage and toxicity. However, more investigations are needed to examine other natural products with JAK/STAT inhibitory effects for glioblastoma and their combinational therapy with other conventional therapies.

## Figures and Tables

**Table 1:** Natural products inhibitory effects on JAK/STAT and related signaling pathways as therapeutic strategy for glioblastoma and other cancers.

Natural compound	Type of natural compound	Chemical formula	Cancer/cell type or animal model studied	Used dosage	Function/mechanism	Ref.
Polyphenols	Resveratrol	C_14_H_12_O_3_	U251 cell line	100 *μ*M	Preventing STAT1 phosphorylation by inhibiting JAK and STAT3 and apoptotic genes induction.	[5, 44, 45]
					ROS generation and induce oxidation-related cellular lesions.	
			LN18 and U87 cell lines	20 *μ*M-40 *μ*M	Reduction of epithelial to mesenchymal transition (EMT), expression of *β*-catenin and decreased the expression of stem cell markers (Twist, Snail, MMP-2, MMP-9, Slug, and Smad).	
	Curcumin	C_21_H_20_O_6_	Non-small-cell lung cancer (NSCLC)	1 *μ*M-20 *μ*M	Improves drug resistance of gefitinib or erlotinib in cancer therapy.	[46–51, 52, 53–56]
			Glioblastoma cells	10 *μ*M-70 *μ*M	Inhibiting tumor microenvironments such as inflammation, angiogenesis, and metastasis. Inhibiting STAT3 through induction of ROS or RANK gene activity by suppressing JAK1, 2/STAT3 phosphorylation through downregulation of MMP-9, c-Myc, Ki-67 and Snail. Suppressing cell proliferation, migration, invasion by inducing G2/M cell cycle arrest.	
	Bergamottin	C_21_H_22_O_4_	Glioblastoma cells such as A549, U87, and U251 cell lines	2 *μ*M and 10 *μ*M	Inhibitor of some cytochrome enzymes, such as P450. Can negatively regulate the cell cycle and activate apoptosis by inhibiting phosphorylation of activated kinases of JAK1, JAK2, C-Src and a SHP-1 and suppressing STAT3 and its downstream products including Bcl-2, Bcl-XL, cyclin D1, COX-2, IAP-1, survivin, and VEGF. Wound-healing, migration, and Matrigel invasion inhibition.	[5, 56, 59–62]
	Bavachin	C_20_H_20_O_4_	Glioblastoma cells	2 *μ*M and 20 *μ*M	Inhibiting of STAT3 transcription by acting as a phytoestrogen. Inhibiting of NF-*κ*B and IL-6-induced STAT3 activity and activating caspases 3 and 9 for stimulating apoptosis. Modulating the expression of phorbol-12-myristate-13-acetate-induced protein 1 (PMAIP1, alternative known as Noxa) and p53 tumor cells survival factors.	[5, 65, 66, 70]
			Microglia, macrophages, and chondrocytes	0.5 *μ*M-10 *μ*M	Inhibiting the expression of iNOS, COX-2, and mPGES-1 and the production of nitric oxide (NO), matrix metalloproteinases (MMPs) and prostaglandin E2 (PGE2). Decreasing the HIF-1*α* activity and regulated transcription of genes related to energy metabolism, such as glucose transporter type 1 (GLUT1) and hexokinase 2 needed for survival of cancer cells.	
	Epigallocatechin gallate (EGCG)	C_22_H_18_O_11_	Glioblastoma cells	—	Inhibiting the progression of tumors by affecting the expression of cell cycle regulatory proteins, inhibiting JAK3/STAT3 signaling, and activating lethal caspases and apoptosis. Telomere shortening, elevating DNA damage through phosphorylation of *γ*-H2AX histone, micronuclei and telomere dysfunction.	[72–76]
			Cholangiocarcinoa (CCA) cells	1 *μ*M-50 *μ*M	Cells' proliferation and migration impairing by STAT1 and STAT3 inactivation in administration of quercetin.	
			Colorectal carcinoma	5 *μ*M	Inhibiting the angiogenesis via the Janus kinase/STAT3/IL-8 pathway in administration of curcumin.	
			PDX mouse model	50 mg/kg		
			Glioblastoma cultures	500 *μ*M	Induction of autophagy and apoptosis.	
	Chalcones	C_15_H_12_O	Glioblastoma stem cells	20 *μ*M-40 *μ*M	Inducing apoptosis by inhibiting STAT3 phosphorylation and activating caspases 8 and 9 and releasing ROS, changing the mitochondrial membrane potential and releasing the cytochrome C. Inhibiting the STAT3 phosphorylation, blocking STAT3 nuclear transport, and attenuating the expression of downstream genes including VEGF, survivin, Bcl-XL, and Bcl-2.	[77–80]
			Prostate cancer cells	20 *μ*g/mL		
	Cardamonin	C_16_H_14_O_4_				
	Garcinol	C_38_H_50_O_6_	Primary and recurrent glioblastoma cells	2.5 *μ*M–40 *μ*M	Decreasing STAT3 and STAT5A protein expression.	[83, 84, 86]
			HCC ells	10 *μ*M-25 *μ*M	Inhibiting the STAT3 acetylation and dimerization, and negatively affects the protein's ability to bind to DNA.	
			Pancreatic cancer cells (BxPC-3)	10 *μ*M and 25 *μ*M	Targeting signaling molecules involved in apoptosis (X-IAP, cIAP, caspase 3/9, PARP cleavage, and NF-ĸB).	
			Breast cancer cell lines MDA-MB-231 and Prostat cancer cell line DU145	10 *μ*M and 25 *μ*M	Decreasing both total and phosphorylated STAT3.	
			U-87 MG and GBM8401 cell lines	2.5 *μ*M–40 *μ*M	Inhibiting proliferation, invasion, and migration of cancer cells by enhancing the hsa-miR-181d/STAT3 and hsa-miR-181d/STAT5A ratios, dose dependently. Downregulation of total as well as pSTAT-3 (Tyr 705) in tumors of garcinol administered mice.	
			U87MG mouse xenograft model MDA-MB-231 xenograft mouse model	1 mg/kg body (intraperitoneal injection) 5 mg/day/animal (oral gavage)		
			Immunocompromised mouse model	1 mg/kg (intraperitoneally)	Reducing glioblastoma tumor growth by attenuating STAT3/5A expression, enhancing the Bax/Bcl-XL apoptotic ratio, and downregulating the Ki-67 proliferation index.	
	Silibinin	C_25_H_22_O_10_	GMB cells and MCF-7 cell line	100 *μ*g/mL	Reducing STAT3 phosphorylation in the presence of JAK2 inhibitors. Downregulating the miR-21 and miR-155 leading to induction of genes related to intrinsic and extrinsic apoptosis.	[10–12]
	Chrysin	C_15_H_10_O_4_	Human umbilical vein endothelial cells (HUVECs)	100 nM-100 *μ*M	Downregulating the soluble IL-6 receptor (IL-6R), glycoprotein 130 (gp130), phosphorylated JAK1 and STAT3 levels, and VEGF. Antitumor activity via direct interaction with multiple molecular targets and modulation of signal transduction pathways involved in cellular metabolism (AMPK/Akt/ERK/PPAR) and inflammation (NF-*κ*B, p38/MAPK, TBK1, and Wnt/*β*-catenin).	[91–94]
			Glioblastoma cells (GBM8901 cells)	25 *μ*M-100 *μ*M	Arresting the cell cycle arrest in the G1 phase due to increasing P21 (waf1/cip1) and activating the P38-MAPK	
	Apigenin	C_15_H_10_O_5_	Glioblastoma cells (U1242 MG and U87 MG cell lines)	10 *μ*M-80 *μ*M	Inducing apoptosis and TNF-*α* and reducing MCL-1 and Bcl-XL through inhibition of JAK1/2and STAT3 phosphorylation. Arresting the cell cycle arrest in the G2/M phase and decreasing the level of Akt, mTOR, ERK, STAT3, and S6K proteins.	[96–101]
			Rat C6 glioma cells	1–100 *μ*mol/L	Altering cytokine profiles, which are important for regulating the immune response.	
	Quercetin	C_15_H_10_O_7_	Multiple cancer types such as glioblastoma	Various concentrations such as 1.5 *μ*M-50 *μ*M	Inducing the apoptosis and arresting phase G1 cell cycle in tumor cells, through its interaction with cell cycle regulators, including cyclin-dependent kinase- (CDK-) 4 and cyclin D1, activating p53, cytochrome c release, and also inducing caspase 9 and caspase 3 release.	[103, 105–107, 126]
			T98G and U87 glioblastoma cell lines	25 *μ*M	Regulating the PI3K/Akt/mTOR signaling pathways, IL-6/STAT3 signaling pathways, modulation of apoptosis-related proteins, altering the intracellular pH (pHi), and MMP-2/9 and fibronectin expression.	
			Glioblastoma mouse model	20 mg/kg (oral gavage or injected intraperitoneally)	Sensitizes glioblastoma to t-AUCB by dual inhibition of Hsp27 and COX-2.	

Terpenoids	Cucurbitacins	C_30_H_42_O_7_	T24 cell line	250 nM-2000 nM	Halting the G2/M phase of the cell cycle by activating caspases 8, 9, and 3 and inhibiting Fas/CD95 as well as STAT3/P53/P21 signaling (cucurbitacin E).	[112–115]
			K562 cells	5 nM-80 nM	Inhibiting STAT3 activation and Raf/MEK/ERK signaling pathways (Cucurbitacin B).	
			U87, U87-EGFR-WT, and U87-EGFRviii glioblastoma cell lines	25 nmol/L-5000 nmol/L	Inhibiting the. Proliferation of glioma cells by decreasing p-JAK1, p-JAK2, p-STAT3, and p-STAT5 and VEGF-induced JAK2 and STAT3 activation levels (cucurbitacin I).	
			U87MG cells	100 nM	Inhibiting the HUVEC tubular formation.	

Steroids	Diosgenin	C_27_H_42_O_3_	C6 glioma cell line Human hepatocellular carcinoma cell lines C3A and HepG2	5 *μ*M-25 *μ*M	Increasing apoptosis, ROS generation, DNA damage, and arrest of the S phase cell cycle. Suppressing the STAT3 activation through JAK1, c-Src, and JAK2. Inhibiting cell proliferation and cell cycle arrest in G0/G1 phase by decreasing the cyclin D protein.	[123–125]
	Dioscin	C_45_H_72_O_16_		25 *μ*M-100 *μ*M		
