# Erratum to “The Role of Natural Products as Inhibitors of JAK/STAT Signaling Pathways in Glioblastoma Treatment”

**DOI:** 10.1155/omcl/9876237

**Published:** 2025-06-26

H. Fahmideh, H. Shapourian, R. Moltafeti, et al., “The Role of Natural Products as Inhibitors of JAK/STAT Signaling Pathways in Glioblastoma Treatment,” *Oxidative Medicine and Cellular Longevity 2022*, no. 1 (2022):1–17, https://doi.org/10.1155/2022/7838583.

In the article titled “The Role of Natural Products as Inhibitors of JAK/STAT Signaling Pathways in Glioblastoma Treatment,” there was a spelling error in author Rasol Molatefi's name in the author list, where Rasol Moltafeti should have read Rasol Molatefi.

Also, there was an error in Table 1. The chemical formula “C_15_H_10_O_5_” has been placed in the Cancer/cell type or animal model studied column instead of the Chemical formula column. The corrected table is shown below:

We apologize for this error.

## Figures and Tables

**Table 1:** Natural products inhibitory effects on JAK/STAT and related signaling pathways as therapeutic strategy for glioblastoma and other cancers.

Natural compound	Type of natural compound	Chemical formula	Cancer/cell type or animal model studied	Used dosage	Function/Mechanism	Ref.
Polyphenols	Resveratrol	C_14_H_12_O_3_	U251 cell line	100 µM	Preventing STAT1 phosphorylation by inhibiting JAK and STAT3 and apoptotic genes induction	[5, 44, 45]
					ROS generation and induce oxidation-related cellular lesions	
			LN18 and U87 cell lines	20–40 µM	Reduction of epithelial to mesenchymal transition (EMT), expression of *β*-catenin and decreased the expression of stem cell markers (Twist, Snail, MMP-2, MMP-9, Slug, and Smad)	
	Curcumin	C_21_H_20_O_6_	Non-small cell lung cancer (NSCLC)	1–20 µM	Improves drug resistance of gefitinib or erlotinib in cancer therapy	[46–51, 53−56, 126]
			Glioblastoma cells	10–70 µM	Inhibiting tumor microenvironments such as inflammation, angiogenesis, and metastasis Inhibiting STAT3 through induction of ROS or RANK gene activity by suppressing JAK1, 2/STAT3 phosphorylation through downregulation of MMP-9, c-Myc, ki-67 and Snail Suppressing cell proliferation, migration, invasion by inducing G2/M cell cyclearrest	
	Bergamottin	C_21_H_22_O_4_	Glioblastoma cells such as A549, U87 and U251 cell lines	2 and 10 µM	Inhibitor of some cytochrome enzymes, such as P450 Can negatively regulate the cell cycle and activate apoptosis by inhibiting phosphorylation of activated kinases of JAK1, JAK2, C-Src and SHP-1 and suppressing STAT3 and its downstream products including Bcl-2, Bcl-xl, cyclin D1, COX-2, IAP-1, survivin, and VEGF Wound-healing, migration and Matrigel invasion inhibition	[5, 56, 59–62]
	Bavachin	C_20_H_20_O_4_	Glioblastoma cells	2 and 20 µM	Inhibiting of STAT3 transcription by acting as a phytoestrogen Inhibiting of NF-*κ*B and IL-6-induced STAT3 activity and activating caspases 3 and 9 for stimulating apoptosis Modulating the expression of phorbol-12-myristate-13-acetate-induced protein 1 (PMAIP1, alternative known as Noxa) and p53 tumor cells survival factors	[5, 65, 66, 70]
			Microglia, macrophages, and chondrocytes	0.5–10 µM	Inhibiting the expression of iNOS, COX-2, and mPGES-1 and the production of nitric oxide (NO), matrix metalloproteinases (MMPs) and prostaglandin E2 (PGE2) Decreasing the HIF-1*α* activity and regulated transcription of genes related to energy metabolism, such as glucose transporter type 1 (GLUT1) and hexokinase 2 needed for survival of cancer cells	
	Epigallocatechin Gallate (EGCG)	C_22_H_18_O_11_	Glioblastoma cells	—	Inhibiting the progression of tumors by affecting the expression of cell cycle regulatory proteins, inhibiting JAK3/STAT3 signaling, and activating lethal caspases and apoptosis Telomere shortening, elevating DNA damage through phosphorylation of *γ*-H2AX histone, micronuclei and telomere dysfunction	[72–76]
			Cholangiocarcinoa (CCA) cells	1–50 µM	Cells' proliferation and migration impairing by STAT1 and STAT3 inactivation in administration of quercetin	
			Colorectal carcinoma	5 µM	Inhibiting the angiogenesis via the Janus kinase/STAT3/IL-8 pathway in administration of curcumin	
			PDX mouse model	50 mg/kg		
			Glioblastoma cultures	500 µM	Induction of autophagy and apoptosis	
	Chalcones	C_15_H_12_O	Glioblastoma stem cells	20–40 µM	Inducing apoptosis by inhibiting STAT3 phosphorylation and activating caspases-8 and 9 and releasing ROS, changing the mitochondrial membrane potential and releasing the cytochrome C Inhibiting the STAT3 phosphorylation, blocking STAT3 nuclear transport, and attenuating the expression of downstream genes including VEGF, survivin, Bcl-XL, and Bcl-2	[77–80]
			Prostate cancer cells	20 μg/mL		
	Cardamonin	C_16_H_14_O_4_				
	Garcinol	C_38_H_50_O_6_	Primary and recurrent glioblastoma cells	2.5–40 µM	Decreasing STAT3 and STAT5A protein expression	[83, 84, 86]
			HCC cells	10–25 µM	Inhibiting the STAT3 acetylation and dimerization, and negatively affects the protein's ability to bind to DNA	
			Pancreatic cancer cells (BxPC-3)	10 and 25 µM	Targeting signaling molecules involved in apoptosis (X-IAP, cIAP, caspase-3, 9, PARP cleavage and NF-*κ*B)	
			Breast cancer cell lines MDA-MB-231 and Prostate cancer cell line DU145	10 and 25 µM	Decreasing both total and phosphorylated STAT3	
			U-87MG and GBM8401 cell lines	2.5–40 µM	Inhibiting proliferation, invasion, and migration of cancer cells by enhancing the hsa-miR-181d/STAT3 and hsa-miR-181d/STAT5A ratios, dose dependently Downregulation of total as well as pSTAT-3 (Tyr 705) in tumors of garcinol administered mice	
			U87MG mouse xenograft model MDA-MB-231 xenograft mouse model	1 mg/kg body (intraperitoneal injection) 5 mg/day/animal (oral gavage)		
			Immunocompromised mice model	1 mg/kg (Intraperitoneally)	Reducing glioblastoma tumor growth by attenuating STAT3/5A expression, enhancing the Bax/Bcl-xL apoptotic ratio, and downregulating the ki-67 proliferation index	
	Silibinin	C_25_H_22_O_10_	GMB cells and MCF-7 cell line	100 µg/mL	Reducing STAT3 phosphorylation in the presence of JAK2 inhibitors Downregulating the miR-21 and miR-155 leading to induction of genes related to intrinsic and extrinsic apoptosis	[10–12]
	Chrysin	C_15_H_10_O_4_	Human umbilical vein endothelial cells (HUVECs)	100 nM–100 µM	Down-regulating the soluble IL-6 receptor (IL-6R), glycoprotein 130 (gp130), phosphorylated JAK1 and STAT3 levels, and VEGF Anti-tumor activity via direct interaction with multiple molecular targets and modulation of signal transduction pathways involved in cellular metabolism (AMPK/Akt/ERK/PPAR) and inflammation (NF-*κ*B, p38/MAPK, TBK1 and Wnt/*β*-catenin)	[91–94]
			Glioblastoma cells (GBM8901 cells)	25–100 µM	Arresting the cell cycle arrest in the G1 phase due to increasing P21 (waf1/cip1) and activating the P38-MAPK	
	Apigenin	C_15_H_10_O_5_	Glioblastoma cells (U1242 MG and U87 MG cell lines)	10–80 µM	Inducing apoptosis and TNF-*α* and reducing MCL -1 and BCL-xl through inhibition of JAK1/2 and STAT3 phosphorylation Arresting the cell cycle arrest in the G2/M phase and decreasing the level of Akt, mTOR, ERK, STAT3, and S6K proteins	[96–101]
			Rat C6 glioma cells	1–100 µmol/L	Altering cytokine profiles, which are important for regulating the immune response	
	Quercetin	C_15_H_10_O_7_	Multiple cancer types such as Glioblastoma	Various concentrations such as 1.5–50 µM	Inducing the apoptosis and arresting phase G1 cell cycle in tumor cells, through its interaction with cell cycle regulators, including cyclin-dependent kinase (CDK)-4 and cyclin D1, activating p53, cytochrome c release, and also inducing caspase-9 and caspase-3 release	[103, 105–107, 127]
			T98G and U87 glioblastoma cell lines	25 µM	Regulating the PI3K/Akt/mTOR signaling pathways, IL-6/STAT3 signaling pathways, modulation of apoptosis-related proteins, altering the intracellular pH (pHi), and MMP-2/-9 and fibronectin expression	
			Glioblastoma mouse model	20 mg/kg (oral gavage or injected intraperitoneally)	Sensitizes glioblastoma to t-AUCB by dual inhibition of Hsp27 and COX-2	

Terpenoids	Cucurbitacins	C_30_H_42_O_7_	T24 cell line	250–2000 nM	Halting the G2/M phase of the cell cycle by activating caspases 8, 9, and 3 and inhibiting Fas/CD95 as well as STAT3/P53/P21 signaling (cucurbitacin E)	[112–115]
			K562 cells	5–80 nM	Inhibiting STAT3 activation and Raf/MEK/ERK signaling pathways (Cucurbitacin B)	
			U87, U87-EGFR-WT and U87-EGFRviii glioblastoma cell lines	25–5000 nmol/L	Inhibiting the. Proliferation of glioma cells by decreasing p-JAK1, p-JAK2, p-STAT3, and p-STAT5 and VEGF-induced JAK2 and STAT3 activation levels (Cucurbitacin I)	
			U87MG cells	100 nM	Inhibiting the HUVEC tubular formation	

Steroids	Diosgenin	C_27_H_42_O_3_	C6 glioma cell line Human hepatocellular carcinoma cell lines C3A and HepG2	5–25 µM	Increasing apoptosis, ROS generation, DNA damage, and arrest of the S phase cell cycle Suppressing the STAT3 activation through JAK1, c-Src, and JAK2 Inhibiting cell proliferation and cell cycle arrest in G0/G1 phase by decreasing the cyclin D protein	[123–125]
	Dioscin	C_45_H_72_O_16_		25–100 µM		

